# Frequency and distribution of 152 genetic disease variants in over 100,000 mixed breed and purebred dogs

**DOI:** 10.1371/journal.pgen.1007361

**Published:** 2018-04-30

**Authors:** Jonas Donner, Heidi Anderson, Stephen Davison, Angela M. Hughes, Julia Bouirmane, Johan Lindqvist, Katherine M. Lytle, Balasubramanian Ganesan, Claudia Ottka, Päivi Ruotanen, Maria Kaukonen, Oliver P. Forman, Neale Fretwell, Cynthia A. Cole, Hannes Lohi

**Affiliations:** 1 Genoscoper Laboratories, Helsinki, Finland; 2 Wisdom Health, Waltham-on-the-Wolds, Leicestershire, United Kingdom; 3 Wisdom Health, Vancouver, Washington, United States of America; 4 MediSapiens Ltd., Helsinki, Finland; 5 Research Programs Unit—Molecular Neurology, University of Helsinki, Helsinki, Finland; 6 Department of Veterinary Biosciences, University of Helsinki, Helsinki, Finland; 7 Folkhälsan Institute of Genetics, Helsinki, Finland; University of Bern, SWITZERLAND

## Abstract

Knowledge on the genetic epidemiology of disorders in the dog population has implications for both veterinary medicine and sustainable breeding. Limited data on frequencies of genetic disease variants across breeds exists, and the disease heritage of mixed breed dogs remains poorly explored to date. Advances in genetic screening technologies now enable comprehensive investigations of the canine disease heritage, and generate health-related big data that can be turned into action. We pursued population screening of genetic variants implicated in Mendelian disorders in the largest canine study sample examined to date by examining over 83,000 mixed breed and 18,000 purebred dogs representing 330 breeds for 152 known variants using a custom-designed beadchip microarray. We further announce the creation of MyBreedData (www.mybreeddata.com), an online updated inherited disorder prevalence resource with its foundation in the generated data. We identified the most prevalent, and rare, disease susceptibility variants across the general dog population while providing the first extensive snapshot of the mixed breed disease heritage. Approximately two in five dogs carried at least one copy of a tested disease variant. Most disease variants are shared by both mixed breeds and purebreds, while breed- or line-specificity of others is strongly suggested. Mixed breed dogs were more likely to carry a common recessive disease, whereas purebreds were more likely to be genetically affected with one, providing DNA-based evidence for hybrid vigor. We discovered genetic presence of 22 disease variants in at least one additional breed in which they were previously undescribed. Some mutations likely manifest similarly independently of breed background; however, we emphasize the need for follow up investigations in each case and provide a suggested validation protocol for broader consideration. In conclusion, our study provides unique insight into genetic epidemiology of canine disease risk variants, and their relevance for veterinary medicine, breeding programs and animal welfare.

## Author summary

Like any human, dogs may suffer from or pass on a variety of inherited disorders. Knowledge of how likely a typical dog is to carry an inherited disorder in its genome, and which disorders are the most common and relevant ones across dog breeds, is valuable for both veterinary care and breeding of healthy dogs. We have explored the largest global dog study sample collected to date, consisting of more than 100,000 mixed breed and purebred dogs, to advance research on this subject. We found that mixed breed dogs and purebred dogs potentially suffer from many of the same inherited disorders, and that around two in five dogs carried at least one of the conditions that we screened for. A dog carrying an inherited disorder is not a “bad dog”–but we humans responsible for breeding selections do need to make sustainable decisions avoiding inbreeding, i.e. mating of dogs that are close relatives. The disease prevalence information we generated during this study is made available online (www.mybreeddata.com), as a free tool for breed and kennel clubs, breeders, as well as the veterinary and scientific community.

## Introduction

Nearly 700 inherited disorders and traits have been described in the domestic dog [1], one of the most genetically well-studied species after humans. Consequently, the wide variety of modern dog breeds represent an excellent biomedical animal model population for identifying disease-related genes that commonly bear relevance for human health as well [2–4]. Knowledge on the underpinnings and genetic epidemiology of canine disorders is also of great importance for veterinary care, and for organizations such as kennel clubs, breed clubs, and dog registries that establish guidelines for sustainable breeding practices. Although the primary focus of current dog registries is on purebred dogs, understanding the genetic epidemiology of inherited disorders in mixed breed dogs is equally relevant. Mixed breed dogs represent a substantial part of the canine population in several countries such as in the United States (US) where 47% of households surveyed in 2014 reported having a mixed breed dog [5].

To date, canine epidemiological studies have largely assessed the prevalence of disorders perceived to be complex in their underpinnings, using endpoint diagnoses based on medical- or insurance records as study phenotypes [6,7]. Such approaches are particularly appropriate for complex disorders, for which no predictive direct genetic testing is typically available. In the present study, we sought to fill a major information gap within canine genetic epidemiology by pursuing comprehensive population screening of genetic variants implicated in Mendelian disorders. In canines, Mendelian disorders are of particular population level relevance due to breed carrier frequencies that can be strikingly high (e.g., ~10–30% [8–10]). We have recently shown in a proof-of-concept study that large-scale DNA screening of disease-implicated variants across purebred dogs is technologically feasible, and represents an efficient diagnostic and research discovery tool for veterinary care, disease research, and breeding [11]. Here we harness that approach to conduct the largest DNA based exploration of the canine disease heritage to date by screening 83,220 mixed breed dogs and 18,102 purebred dogs of varying breed and geographical backgrounds for 152 known Mendelian disease variants, covering the majority of the current 212 canine entries in the Online Mendelian Inheritance in Animals (OMIA) [1] database.

We present descriptive statistics on the proportion of dogs in the general population carrying an inherited Mendelian disorder in their genome, and broadly characterize the relative prevalence of canine Mendelian disease variants in mixed breed dogs and in a representative sample of purebreds. In an effort to make the generated, and future, big data easily available to the canine health research community, we create a public canine inherited disorder prevalence database, MyBreedData (www.mybreeddata.com). In conjunction with the conducted population screening we discovered presence of several disease variants in additional breeds in which they were previously undescribed, providing novel insight into the biological mechanisms underlying disease predisposition for breed health research and management. Taken together, our study provides unique insights into the genetic epidemiology of disease risk variants in both mixed breed and purebred dogs, and their relevance for veterinary care, breeding programs, and animal welfare.

## Results

### High overall prevalence of genetic disease variants in both mixed breed and purebred dogs

**Fig 1:**
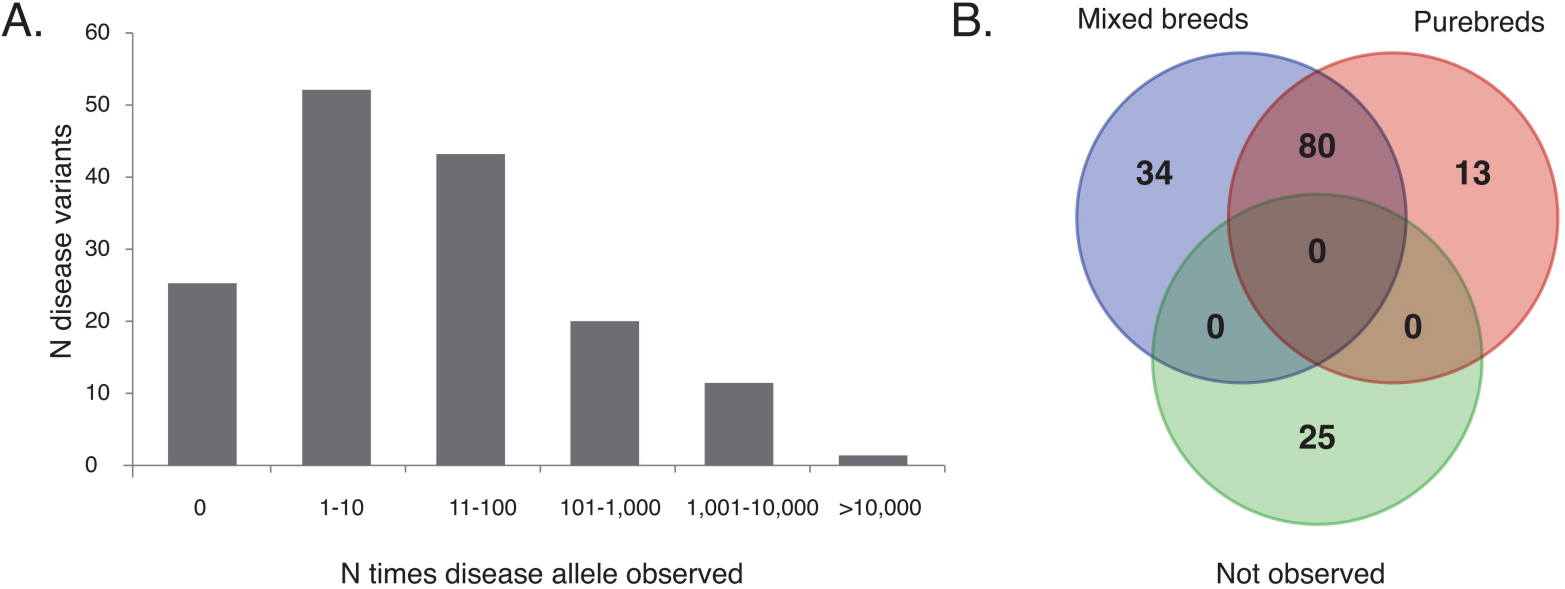
Presence and distribution of 152 Mendelian disease alleles in 101,322 dogs. (A) Frequency distribution of the tested variants highlights a majority of individual disease alleles as absent or rare on a general population level, and a subset of more prevalent disorders. (B) Venn diagram summary of the distribution of disease variants across mixed breed, and purebred dogs. The majority of disorders (N = 80) were observed at least once in both mixed breed and purebred dogs. Other disorders were exclusively observed in either group. Twenty-five of the studied disease variants were not observed in any dog studied. For details on specific disorders and their allele counts, please refer to S3 and S4 Tables.

A total of 101,427 animals were included in the present study (83,220 mixed breed dogs; 18,102 purebred dogs representing 330 different breeds; and 105 wild canids; S1 Table). The vast majority of all dogs (N = 96,514) were genotyped for a full set of 152 genetic variants underlying canine inherited disorders (S2 Table). Of the 152 screened disease variants, 127 (83.6%) were observed in at least one dog in the study population. The majority of the disease alleles (62.5%; N = 95) were encountered between 1–100 times (Fig 1A). Notably, a subset consisting of the thirty most frequently observed disease alleles accounted for 96.1% of all disease variants observed in the study sample.

We initially focused on a descriptive quantification of canine genetic disease allele presence in the subset of dogs (N = 96,514; 83,220 mixed breed dogs and 13,294 purebred dogs from 303 breeds) that had been genotyped for a full set of 152 known disease variants. We found that 40.5% of all dogs carried at least one of the tested disease variants in their genome in either hetero- or homozygous state (Table 1). The maximum number of genetic disease variants observed in any individual dog was 5.

**Table 1:** Distribution of Mendelian disease variant presence based on 152 known mutations genotyped in 96,514 dogs.

		N different disease variants present^a^						
		0	1	2	3	4	5	Total
**All dogs**	**N**	57,454	30,581	7,336	1,048	89	6	96,514
	**%**	59.53	31.69	7.60	1.09	0.09	0.01	100.00
**Mixed breed dogs**	**N**	48,413	27,082	6,660	978	81	6	83,220
	**%**	58.17	32.54	8.00	1.18	0.10	0.01	100.00
**Combined purebred sample**	**N**	9,041	3,499	676	70	8	0	13,294
	**%**	68.01	26.32	5.09	0.53	0.06	0.00	100.00

^a^ in either hetero- or homozygous state.

### Mixed breed and purebred dogs share the same common inherited disease variants

Most tested disease variants were encountered in both mixed breed and purebred dogs (N = 80) while others were exclusively observed in one of the groups (Fig 1B; S3 Table). Comparison of the two study populations most importantly confirms some disease variants as virtually completely breed specific (e.g., Lagotto Romagnolo [N = 824] juvenile epilepsy [breed carrier frequency 28.3%; absent in other dogs], Coton de Tulear [N = 253] canine multifocal retinopathy 2 [breed carrier frequency 15.8%; absent in other dogs], Standard Poodle [N = 184] neonatal encephalopathy with seizures [breed carrier frequency 16.8%; only observed in one mixed breed dog], cystinuria type I-A of Newfoundlands [N = 67; breed carrier frequency 14.9%; only observed in one mixed breed dog], glycogen storage disease type IIIa of Curly Coated Retrievers [N = 156; breed carrier frequency 8.3%; only observed in one mixed breed dog], mucopolysaccharidosis type VII of Brazilian Terriers [N = 87; breed carrier frequency 25.3%; absent in other dogs], and pyruvate dehydrogenase phosphatase deficiency 1 of Clumber and Sussex Spaniels [N = 69; breed carrier frequency 30.4%; absent in other dogs]). Conversely, other disease variants such as neuronal ceroid lipofuscinosis 5 of Border Collies and pyruvate kinase deficiency of Beagles are still segregating in mixed breed dogs but potentially eradicated from their original discovery pure breeds (carrier frequencies in mixed breed dogs 0.12% and 0.01%, respectively, but not observed in 125 Border Collies or in 119 Beagles). While most canine Mendelian disorders are recessive, the dataset included genotypes for nine variants characterized as following an autosomal dominant mode of inheritance (S2 Table). The three dominant disease variants repeatedly observed in both mixed breed dogs and in the combined purebred sample have been associated with primary hereditary cataract [12], craniomandibular osteopathy [13], and cystinuria type II-A [14].

We subsequently identified the most common individual canine inherited disease variants present in the study sample (N = 101,322 dogs after excluding wild canids). The twelve most frequent disease variants in mixed breed dogs and in the combined population of purebred dogs were essentially the same (Table 2), with the list consisting of likely ancient variants known to be present in multiple breed groups. These variants predispose to disorders such as degenerative myelopathy (DM; [15]), progressive rod-cone degeneration (*prcd*-PRA; [16]), hyperuricosuria (HUU; [17]), collie eye anomaly (CEA; [18]), exercise-induced collapse (EIC; [19]) and von Willebrand’s disease type 1 (vWD 1; [20]). A complete prevalence-based ranking list of all examined 152 disease variants with full allele and genotype counts is provided as S4 Table.

**Table 2:** Top 30 most frequently observed disease variants in mixed breed dogs.

			Mixed breed dogs		Combined purebred study sample	
Tested disease variant	OMIA^a^ entry	Breed(s) variant was previously characterized in	Rank	Disease allele frequency [%]	Rank	Disease allele frequency [%]
Degenerative Myelopathy (DM)	000263–9615	>120 breeds	**1**	7.771	**1**	5.414
Cone-Rod Dystrophy (cord1-PRA/crd4)^b^	001432–9615	>5 breeds	**2**	3.664	**3**	1.519
Progressive Rod-Cone Degeneration (*prcd*-PRA)	001298–9615	>30 breeds	**3**	3.418	**2**	1.746
Hyperuricosuria (HUU)	001033–9615	>30 breeds	**4**	2.155	**5**	1.319
Collie Eye Anomaly (CEA)	000218–9615	>10 breeds	**5**	1.600	**6**	1.080
Exercise-Induced Collapse (EIC)	001466–9615	>10 breeds	**6**	1.131	**7**	1.005
Multidrug Resistance 1 (*MDR1* gene variant)	001402–9615	>15 breeds	**7**	1.046	**8**	0.989
von Willebrand's Disease Type 1 (vWD 1)	001057–9615	>20 breeds	**8**	0.768	**4**	1.460
Golden Retriever Ichthyosis	001588–9615	Golden Retriever	**9**	0.710	**12**	0.699
Primary Lens Luxation (PLL)	000588–9615	>20 breeds	**10**	0.613	**9**	0.771
von Willebrand's Disease Type 2 (vWD 2) ^c^	001339–9615	>10 breeds	**11**	0.595	**10**	0.708
Factor VII Deficiency	000361–9615	>15 breeds	**12**	0.487	**11**	0.707
Neuronal Ceroid Lipofuscinosis 4A (NCL4)	001503–9615	American Staffordshire Terrier	**13**	0.397	**28**	0.094
Hypocatalasia or Acatalasemia	001138–9615	Beagle	**14**	0.264	**27**	0.097
Prekallikrein Deficiency	000819–9615	Shih Tzu	**15**	0.206	**32**	0.075
Primary Open Angle Glaucoma (POAG)	001870–9615	Norwegian Elkhound	**16**	0.183	**17**	0.321
Cone-Rod Dystrophy 1 (crd1)	001674–9615	American Staffordshire Terrier	**17**	0.172	**51**	0.026
Skeletal Dysplasia 2 (SD2)	001772–9615	Labrador Retriever	**18**	0.151	**21**	0.163
Primary Hereditary Cataract (PHC)	001758–9615	Australian Shepherd	**19**	0.137	**24**	0.123
Golden Retriever Progressive Retinal Atrophy 2 (GR_PRA 2)	001984–9615	Golden Retriever	**20**	0.136	**52**	0.026
Centronuclear Myopathy (CNM)	001374–9615	Labrador Retriever	**21**	0.121	**25**	0.117
Craniomandibular Osteopathy (CMO)	000236–9615	Scottish Terrier, West Highland White Terrier, Cairn Terrier	**22**	0.114	**14**	0.362
Cystinuria Type II-A	001879–9615	Australian Cattle Dog	**23**	0.105	**54**	0.026
Persistent Müllerian Duct Syndrome (PMDS)	000791–9615	Miniature Schnauzer	**24**	0.096	**31**	0.080
Protein Losing Nephropathy (PLN); *NPHS1* gene variant	001326–9615	Irish Soft Coated Wheaten Terrier, Airedale Terrier	**25**	0.078	**16**	0.338
Hereditary Nasal Parakeratosis (HNPK)	001373–9615	Labrador Retriever	**26**	0.075	**22**	0.160
Imerslund-Gräsbeck Syndrome (IGS)	001786–9615	Border Collie	**27**	0.075	**54**	0.026
Neuronal Ceroid Lipofuscinosis 5 (NCL5)	001482–9615	Border Collie	**28**	0.060	**94**	0.000
Macrothrombocytopenia	001001–9615	Norfolk Terrier, Cairn Terrier	**28**	0.060	**15**	0.350
Primary Hereditary Cataract (PHC)	001758–9615	Boston Terrier, Staffordshire Bull Terrier, French Bulldog	**30**	0.060	**35**	0.064

^a^ Online Mendelian Inheritance in Animals (http://omia.angis.org.au)

^b^ Note variant originally characterized with autosomal recessive inheritance, but effect influenced by modifier variants [21,22]

^c^ Note causality of variant under question [23]

### A novel canine disease variant prevalence database—MyBreedData

This study generated an extensive dataset on the prevalence of known breed-relevant disorders across a wide variety of purebreds. The full genotype raw data for all animals is available from the Dryad Digital Repository (https://doi.org/10.5061/dryad.dd91b). However, to make this breed-specific genotype frequency data publicly available and easily accessible for breed health research purposes, we have let it serve as a foundation for a newly created online resource called the MyBreedData Canine Inherited Disorder Prevalence Database, accessible at http://www.mybreeddata.com. The database features the possibility to search for disease variant genotype frequencies by breed or disorder, and is amenable to regular updates to keep the information current.

### Genotyping-based evidence for hybrid vigor in mixed breed dogs

**Fig 2:**
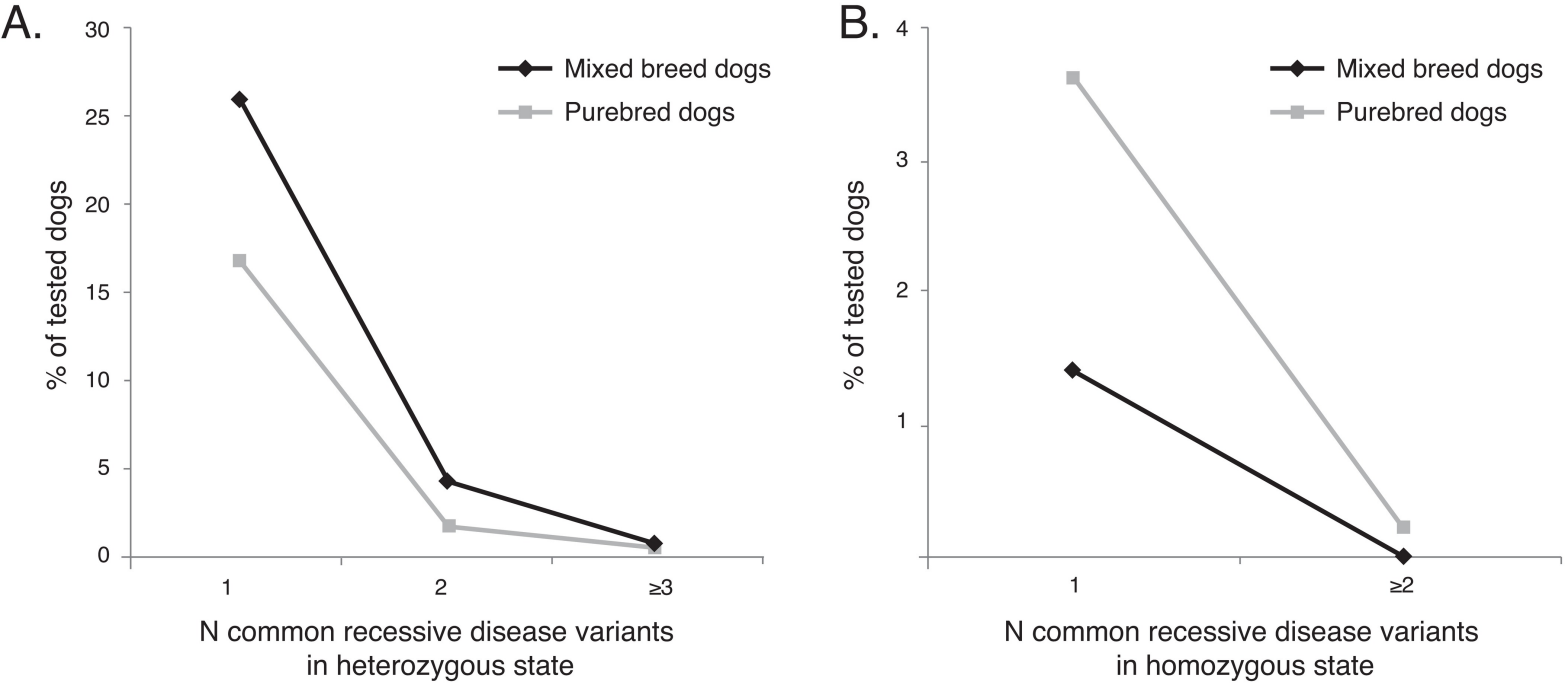
Patterns of common recessive disease variant presence in 96,673 dogs. (A) Comparison of 83,220 mixed breed dogs and 13,453 purebred dogs revealed that mixed breed dogs were significantly more likely to carry one or several of nine examined common largely recessive disease variants in the heterozygous state. (B) Conversely, dogs of the combined purebred sample were more likely to be genetically affected for one of the examined disorders, i.e. carry at least one recessive disease variant in the homozygous state.

Actual genotype data based on molecular measurements across multiple disorders allows direct analyses exploring the notion of whether mixed breed dogs shows signs of hybrid vigor for recessive disorders. Conversely, one can search for signs of whether the general purebred population shows an increased likelihood for inheriting two copies of the same recessive disease variant, as statistically expected in closed breeding populations. We restricted the analysis to nine well known disease variants widespread across both the mixed and purebred populations that in clinical practice most closely follow an autosomal recessive mode of inheritance, and are associated with phenotype effects that have late onset or remain largely undiagnosed without detailed clinical examinations of the dog: DM, *prcd*-PRA, HUU, CEA, vWD 1, EIC, multidrug resistance 1 (MDR1; [24]), primary lens luxation (PLL; [25]), and factor VII (FVII) deficiency [26]. Characteristics of co-dominant inheritance are acknowledged for MDR1, but the heterozygous state is typically not associated with a life-threatening deficiency. Genotypes from 96,673 dogs (83,220 mixed breed and 13,453 purebred dogs) were available for the analysis. We first examined whether mixed breed and purebred dogs differed in the number of disease variants carried in the heterozygous state, and observed that mixed breed dogs were 1.6 times more likely than purebreds (30.3% vs. 18.4% of dogs, respectively) to be carriers of at least one of the nine recessive disease variants included in the analysis. The distribution of the number of disease variants carried in the heterozygous state differed significantly between mixed breed dogs and the combined purebred sample, with a higher ratio of mixed breed dogs being carriers of the common analyzed disease risk alleles (Fig 2A; χ^2^ (df = 3) = 842.78, P < 10^−4^). However, when we compared the groups for the number of common recessive disease variants carried in the homozygous state, an opposite pattern emerged (Fig 2B). Purebred dogs were 2.7 times more likely than mixed breed dogs to be genetically affected for at least one of the common recessive disorders (3.9% vs. 1.4% of dogs, respectively); χ^2^ (df = 2) = 488.48, P < 10^−4^).

### Clinical signs of canine inherited disorders are also observed in mixed breed dogs

It is important to understand how the identified genetic risk variants will clinically manifest in dogs of mixed breed ancestry in order to provide proper counseling to clinicians and dog owners. We therefore pursued clinical validation of disease variants not previously described in dogs of mixed breed ancestry by interviewing owners. These interviews sought to determine if a dog genetically at risk of developing a disorder exhibited clinical manifestations similar to those expected for the condition in the affected purebred population. We are continuously collecting this type of information and, within the scope of this study, recount the following case studies as examples.

A first clinical confirmation involved a 1.5-year old female spayed mixed breed dog of Labrador Retriever/Rat Terrier/Siberian Husky/Golden Retriever/Australian Shepherd/mix ancestry that was determined to be genetically at risk for exercise-induced collapse (EIC; [19]) as described in several retriever and sporting breeds. On interview, the owner reported the dog having experienced collapsing episodes during exercise/play on two separate occasions that had prompted them to visit an emergency clinic where a medical cause was not identified. The owner was relieved to receive a genetic explanation enabling informed management of the condition.

A second clinical confirmation involved an approximately 4-year-old male neutered Miniature Poodle/Yorkshire Terrier/mix dog that also had some distant matches to the Parson Russell Terrier (greater than three generations ago). This dog carried two copies of the recessive spinocerebellar ataxia (*CAPN1* gene) mutation originally identified in Parson Russell and Jack Russell Terriers [27]. The dog’s owner provided video evidence and confirmed that this dog displayed the clinical signs associated with spinocerebellar ataxia including an abnormal hind limb gait, uncoordinated movements, and impaired balance resulting in frequent falls; a feature the owner had always attributed to the dog “just being clumsy.” The dog was not reported to have the more severe clinical manifestations of the disease including seizures, exercise intolerance or behavioral changes.

A third mixed breed dog evaluated was an 11-year-old male neutered Chow Chow/Chinese Shar Pei/Collie/Miniature Poodle/mix dog that carried two copies of the skeletal dysplasia 2 (SD2; *COL11A2* gene) mutation originally identified in Labrador Retrievers [28]. While this dog did not show evidence of a recent Labrador Retriever ancestor, the client was not surprised to learn of the mutation finding given they anticipated seeing a typical chondrodysplastic breed, such as the Dachshund or Basset Hound, in his ancestral makeup given his appearance. Images received from the owner confirmed that he did exhibit disproportionate dwarfism resembling the more typical breed-defining chondrodysplasia variant in the *FGF4* gene [29], despite testing negative for this mutation.

Notably, of the aforementioned dogs, the *CAPN1* and *COL11A2* affecteds had higher than expected homozygosity levels for mixed breed dogs indicating that they may have been the result of matings between close relatives, which could explain the presence of two copies of a rarer mutation on a mixed breed background.

Finally, numerous accounts of mixed breed dogs with one copy of the multidrug sensitivity (MDR1) mutation [24] were also collected. One such account related to a mixed breed dog (62.5% Beagle / 25% Australian Shepherd / 12.5% mix) whose owner was a veterinary clinician and reported the dog exhibiting clinically noticeable mental alterations for several days post-anesthesia before making a full recovery. More broadly, the collected accounts related to MDR1 describe an appreciably delayed recovery from anesthetic procedures that include the use of acepromazine and butorphanol as part of the anesthetic protocol. Processing and elimination of both medications are known to be affected by the MDR1 mutation. Owners and clinicians reported that these dogs require up to four days to return to normal levels of activity and mental acuity compared to dogs without the MDR1 mutation who receive the same anesthetic protocol and typically return to normal activities by the following day.

### Disease variants discovered in additional purebreds

**Fig 3:**
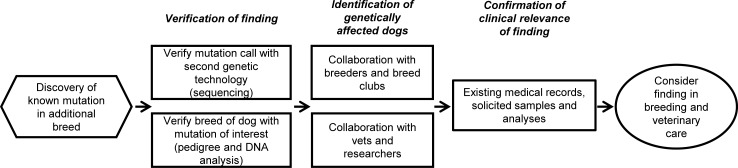
A proposed standard operating procedure for handling mutation discoveries in additional breeds.

In addition to breeds in which particular disease mutations are known to exist, we identified mutations present in other purebred populations in which they had not been previously reported in the peer-reviewed scientific literature. Following the routine operating procedure adopted by our organization (Fig 3), we have pursued genetic and clinical validation of several discoveries. We validated the genetic presence of 22 variants in a total of 81 breeds (Table 3). Notably, several additional breeds genetically carry published risk variants for CEA, EIC, FVII deficiency, HUU, *prcd*-PRA, and vWD 1. The spectrum of discoveries made includes both logical findings in light of breed history (e.g., hereditary footpad hyperkeratosis (HFH) of Kromfohrländers and Irish Terriers [30] now discovered in Bedlington-, Welsh-, and German Hunting Terriers), as well some more surprising ones. For an example, we unexpectedly discovered Australian Shepherd-type primary hereditary cataract [12] carried by American (but no European) Miniature Pinschers, and the P2RY12 defect-related bleeding disorder variant originally characterized in Greater Swiss Mountain Dog [31] in Bichon Frise. Notably, we also observed 1 out of 13 tested dingos carrying degenerative myelopathy, while none of the other tested canine disease variants were found in gray wolves, coyotes or dingos.

**Table 3:** Summary of disease variant findings in additional breeds.

Tested disease variant	OMIA^a^ entry	Breed(s) variant was previously characterized in	Additional breed(s) with carrier frequency
Bleeding disorder due to P2RY12 defect	001564–9615	Greater Swiss Mountain Dog	Bichon Frise: 6.9% (4/58)
Collie Eye Anomaly (CEA)	000218–9615	>10 breeds	Australian Kelpie: 7.5% (6/80)
			Chinook: 0.7% (1/151)
			Jack Russell Terrier: 3.8% (4/105)
			Parson Russell Terrier: 1.2% (3/243)
			Koolie: 41% (16/39)
			Lapponian Herder: 5% (1/20)
			Tamaskan Dog: 3.5% (3/86)
Craniomandibular Osteopathy (CMO)	000236–9615	Scottish Terrier, West Highland White Terrier, Cairn Terrier	American Staffordshire Terrier: 0.4% (1/258)
			Skye Terrier: 20% (2/10)
Cystinuria, Type II-A	001879–9615	Australian Cattle Dog	Border Collie: 1.9% (2/106)
			Koolie: 7.7% (3/39)
Exercise-Induced Collapse (EIC)	001466–9615	>10 breeds	Coton de Tulear: 2.4% (6/253)
			Parson Russell Terrier: 3.3% (8/243)
			Rhodesian Ridgeback: 2.5% (6/236)
Factor VII Deficiency	000361–9615	>15 breeds	Basset Hound: 2.3% (1/44)
			Catahoula Leopard Dog: 3.8% (1/26)
			Dachshund—Miniature Shorthaired: 2.2% (1/45)
			English Foxhound: 4.3% (1/23)
			German Shorthaired Pointer: 8.1% (5/62)
			Harrier: 16.7% (4/24)
			Redbone Coonhound: 33.3% (3/9)
			Whippet: 1.1% (1/91)
Golden Retriever Ichthyosis	001588–9615	Golden Retriever	Alaskan Husky: 20% (1/5)
			Rhodesian Ridgeback: 0.4% (1/236)
Hereditary Footpad Hyperkeratosis (HFH)	001327–9615	Kromfohrländer, Irish Terrier	Bedlington Terrier: 21.8% (12/55)
			German Hunting Terrier: 11.1% (1/9)
			Welsh Terrier: 3.3% (2/61)
Hyperuricosuria (HUU)	001033–9615	>30 breeds	Airedale Terrier: 3.6% (2/56)
			Anatolian Shepherd Dog: 5% (1/20)
			Boston Terrier: 2.9% (2/70)
			Canaan Dog: 5.9% (1/17)
			English Setter: 2.0% (1/49)
			Greater Swiss Mountain Dog: 12.3% (7/57)
			Maltese: 1.4% (1/73)
			Maremma and Abruzzes Sheepdog: 9.1% (2/22)
Hypocatalasia or Acatalasemia	001138–9615	Beagle	English Foxhound: 4.3% (1/23)
			Harrier: 25% (6/24)
			Poodle—Miniature (AKC size standard): 1.2% (1/83)
			Treeing Walker Coonhound: 4.9% (2/41)
Macrothrombocytopenia	001001–9615	Norfolk Terrier, Cairn Terrier	Chihuahua: 1.9% (3/160)
			Danish-Swedish Farmdog: 5.9% (7/118)
			Kerry Blue Terrier: 19.4% (7/36)
			Kritikos Lagonikos: 11.9% (5/42)
			Maremma and Abruzzes Sheepdog: 13.6% (3/22)
			Parson Russell Terrier: 0.9% (1/110)
			West Highland White Terrier: 1% (2/193)
Neuronal Ceroid Lipofuscinosis 8 (NCL8)	001506–9615	English Setter	Danish-Swedish Farmdog: 0.9% (1/118)
Osteochondrodysplasia	001315–9615	Miniature Poodle	Papillon: 12.9% (11/85)
Prekallikrein Deficiency	000819–9615		American Hairless Terrier: 6.3% (2/32)
Primary Hereditary Cataract (PHC)	001758–9615	Australian Shepherd	Miniature Pinscher: 6.9% (4/58)
			Bull Terrier: 1.9% (1/53)
Primary Open Angle Glaucoma (POAG)	001870–9615	Beagle	East-Siberian Laika: 15.2% (5/33)
Progressive Retinal Atrophy (PAP1_PRA)	000830–9615	Papillon, Phalene	Brussels Griffon: 2.2% (1/45)
Progressive Rod-Cone Degeneration (*prcd*-PRA)	001298–9615	>30 breeds	Biewer Terrier: 3.4% (1/29)
			Black Russian Terrier: 6.7% (2/30)
			Bolognese: 7.1% (1/14)
			Coton de Tulear: 9.7% (18/186)
			Field Spaniel: 17.7% (6/34)
			Jack Russell Terrier: 1.7% (1/60)
			Japanese Chin: 2.1% (1/48)
			Lagotto Romagnolo: 2.7% (12/452)
			Lancashire Heeler: 11.1% (1/9)
			Manchester Terrier—Toy: 11.4% (4/35)
			Mi-ki: 1.4% (1/69)
			Norrbottenspitz: 6.3% (1/16)
			Plott: 24% (6/25)
			Pomeranian: 1.3% (1/76)
			Portuguese Podengo—Miniature: 14.3% (5/35)
			Puli: 2.8% (1/36)
			Russian-European Laika: 20% (1/5)
			Serbian Hound: 100% (1/1)
			Tibetan Terrier: 2% (1/51)
			Xoloitzcuintle: 6.7% (1/15)
Protein Losing Nephropathy (PLN); *NPHS1* gene variant	001326–9615	Irish Soft Coated Wheaten Terrier, Airedale Terrier	Danish-Swedish Farmdog: 22.9% (27/118)
Skeletal Dysplasia 2 (SD2)	001772–9615	Labrador Retriever	American Cocker Spaniel: 2.6% (2/77)
			Cavalier King Charles Spaniel: 6.3% (7/112)
			Sussex Spaniel: 11.8% (2/17)
von Willebrand's Disease Type 1 (vWD 1)	001057–9615	>20 breeds	Australian Shepherd: 5.5% (8/146)
			Australian Silky Terrier: 2.6% (1/39)
			Cairn Terrier: 9.7% (6/62)
			German Spitz: 46.3% (19/41)
			Great Dane: 2.7% (2/73)
			Pomeranian: 13.2% (10/76)
			Prague Ratter: 100% (1/1)
			Pug: 16% (8/50)
			Puli: 25% (9/36)
			Schipperke: 25.5% (12/47)
			Volpino Italiano: 7.7% (2/26)
von Willebrand's Disease Type 2 (vWD 2)	001339–9615	>10 breeds	American Cocker Spaniel: 4.2% (3/71)
			Australian Stumpy Tail Cattle Dog: 40% (2/5)
			Boykin Spaniel: 37.5% (3/8)
			Canaan Dog: 35.3% (6/17)
			Chihuahua: 5% (8/160)
			German Spitz: 4.9% (2/41)
			Koolie: 2.6% (1/39)
			Poodle—Miniature (AKC size standard): 4.8% (4/83)
			Poodle—Standard (AKC size standard): 5.4% (3/56)
			Poodle—Standard (FCI size standard)—Black, brown and white: 2.9% (1/34)
			Spinone Italiano: 11.3% (6/53)
			Xoloitzcuintle: 6.7% (1/15)

^a^ Online Mendelian Inheritance in Animals (http://omia.angis.org.au)

Through case studies, we further aimed to establish clinical relevance for as many of the genetic discoveries made through panel screening in additional breeds as possible. We have previously reported on the genetic discovery of vWD type 1 in the Kromfohrländer breed [11], and within the context of this study pursued further validation of the breed discovery. A blood plasma sample from a female Kromfohrländer genetically affected for vWD type I was submitted for vWF associated antigen (vWF:Ag) testing, with the results indicating decreased vWF levels of 22% compared to the reference range indicated by the laboratory service provider (55–150%), and a control sample from a genetically “clear” dog of the same breed submitted simultaneously for testing (96%). Given the large number of additional breeds (Table 3 and [11]) discovered to tentatively carry vWD type 2, we also submitted samples from dogs homozygous for the originally published putative variant (*VWF* c.4937A>G; [32]) for vWF:Ag testing (Table 4). The results of all tested dogs were either borderline or within the reference range specified by the clinical testing laboratory, lending further support to the notion [23] that the originally published variant is in fact not the causal variant in the gene.

**Table 4:** Summary of von Willebrand factor (VWF) measurements in dogs at putative genetic risk.

Dog	Breed	Tested variant	Genotype	Plasma vWF:Ag [reference range]
1	Kromfohrländer	vWD type 1 (*VWF* c.7437G>A)	A/A	22% [55–150%]
2	Barbet	vWD type 2 (*VWF* c.4937A>G)	G/G	89% [48–172%]
3	Chinese Crested Dog	vWD type 2 (*VWF* c.4937A>G)	G/G	48% [55–150%]
4	Chinese Crested Dog	vWD type 2 (*VWF* c.4937A>G)	G/G	59% [55–150%]
5	Norwegian Elkhound, Grey	vWD type 2 (*VWF* c.4937A>G)	G/G	149% [70–180%]

The widespread progressive rod-cone degeneration (*prcd*-PRA) mutation is generally thought to have a highly penetrant effect, making genetic testing relevant across breeds [16]. Within the scope of this study, we were able to identify three Miniature Portuguese Podengos and two Bolognese dogs with both an ophtalmological examination result indicating PRA (Finnish Kennel Club Registry Database [33]), and DNA sample availability at the Canine DNA Bank of the University of Helsinki/Folkhälsan Research Center, Finland. All three Podengos, and one of two Bologneses were confirmed by genetic testing to be homozygous for the *prcd*-PRA mutation, further supporing the penetrance of the variant across breeds. Notably, the Podengos had been diagnosed with PRA at 6–9 years of age, suggesting late disease onset in the breed.

Breed clubs representing Bedlington-, Welsh-, and German Hunting Terriers were contacted regarding the hereditary footpad hyperkeratosis (HFH) risk variant discovery made in the breeds. Breeders of Bedlington Terriers indicated that the phenotype is well known to exist in the breed, albeit with a limited number of cases reported since the year 2000. Anecdotal evidence provided by breeders suggested some individuals having privately pursued genetic testing for the Irish Terrier / Kromfohrländer risk variant in the past, obtaining an “affected” test result in dogs manifesting the phenotype. To the best of our knowledge, such observations have not been published and although we are unable to directly confirm this we find it plausible that the same risk variant for HFH is shared and relevant across multiple terrier breeds.

Increased risk for urate urolithiasis conveyed by a well known hyperuricosuria (HUU) associated variant in the *SLC2A9* gene was reported in several breeds lacking recent common ancestry [9]. Therefore, awareness and genetic testing for the variant is likely to be relevant in additional discovered breeds (Table 3) where the mutation frequency is reasonably high (e.g., Greater Swiss Mountain Dog). Outside of our primary study population, we further report the discovery of a high carrier frequency of 31%, and genetically affected frequency of 8.5%, in the Schapendoes breed. Through owner-submitted medical records, we confirm at least one Schapendoes case showing urine sediment containing urate crystals, further highlighting the diagnostic value of genetic testing for the *SLC2A9* variant across breeds.

## Discussion

Purebred canines offer a model population in which, among other well-documented benefits for genetic studies, the carrier frequencies of several Mendelian disorders are high [2,3]. Consequently, comprehensive DNA screening for Mendelian disorders can have immediate practical relevance for breeding selections and veterinary care. Simultaneously, panel screening of mutations provides an efficient platform for the initial discovery of disease variants in additional breeds—discoveries that are typically best explained by breed history in combination with breeding practice as shown by our previous evaluation of 93 disease-associated variants in approximately 7000 purebred dogs representing 230 breeds [11]. In the present study, we further extended our work to cover 152 disease-implicated genetic variants in over 18,000 purebred dogs from 330 breeds. Concurrently, we provide the first ever snapshot of the disease heritage of mixed breed dogs by examination of the same risk variants in over 80,000 mixed breed dogs. To our knowledge, the examined population represents by far the largest canine sample genotyped in a single study to date.

Like every human being, every dog is likely to carry a number of deleterious mutations in its genome. The patterns of predicted deleterious genetic variation shaping the dog genome have been explored in the past [34]. For the first time, our study covering a wide variety of breed backgrounds provides a direct genetic screening-based quantitative estimate of the ratio of dogs in the general canine population carrying a risk variant for Mendelian disorders: around two in five dogs (40.5%). We note as a general limitation that any estimate of this type is restricted to consideration of the population of hitherto identified disease variants included in the screening. Notably, although the majority of the tested variants were observed at least once in the study sample, around thirty common disorders account for the overwhelming majority of the disease alleles observed. Such information could potentially be used to guide veterinary education and pet care, and training in genetic counseling. At the other end of the spectrum, we note that several disease variants described in the literature are likely extremely rare in, or have been eradicated from, the general dog population (S3 Table, S4 Table). Careful validation of all individual disorder assays with known genotype control samples or synthetic oligonucleotide controls was performed, reducing the likelihood that a disorder would have been undetected due to technical issues.

Our data further reveals that the most common genetic disease variants are essentially the same in both the mixed and purebred populations. The finding is logical in light of all of the most prevalent disease variants having been previously described in multiple different pure breeds (Table 2). Such widespread disease variants are likely to be ancient in origin, predating or having occurred early during the formation of modern dog breeds. Notably, with the exception of one dingo carrying the most widespread variant of them all–degenerative myelopathy (DM)–the tested mutations were absent from the modern wild canids screened. The prevalence of many disorders in mixed breed dogs may be explained by the popularity of the breed the disease mutation was originally discovered in (e.g., Labrador Retriever [American Kennel Club [35] popularity ranking 1^st^]; Golden Retriever [3^rd^]; Beagle [5^th^], Australian Shepherd [16^th^], Miniature Schnauzer [17^th^], or Shih Tzu [20^th^]) and the tendency of a breed to be observed in the background of the studied US mixed breed dogs (e.g., American Staffordshire Terrier, Australian Cattle Dog, Beagle, and Golden Retriever; as indicated in the Materials and methods section). Disease alleles are likely to abide in the mixed breed population due to random mating and reduced selective pressure, as increased heterozygosity reduces the likelihood of recessive disease allele homozygous dogs at risk of showing signs of a rare condition, and many common conditions additionally have late onset, a minor effect on reproductive fitness, or a minor clinical phenotype. As expected, dominant disease alleles encountered in our dataset were described as exhibiting incomplete penetrance, adult age of onset, or clinical signs that may not be evidently associated with a specific genetic cause, facilitating their segregation in the population. In purebreds, disease allele presence may be influenced to a higher extent by increased awareness of breed-relevant health issues combined with active negative selection and restrictions imposed by breed organizations, such as mandatory DNA testing for breeding dogs.

Statistical comparisons between the mixed breed and purebred populations are inherently sensitive to challenges in defining “ideal” breed contributions for a representative global purebred sample, as well as the availability of samples from individual breeds where bias may be introduced by attitude differences between breed clubs regarding participation in DNA testing activities. Nevertheless, comparison between the mixed breed sample and our purebred sample–a unique collection of dogs from a wide variety of more than 300 breed backgrounds (median contribution of any individual breed to the combined purebred sample was only 0.19%)–provides important value in confirming complete, or virtually complete, breed-specificity for several disease variants. Such breed-specific mutations are likely to have arisen fairly recently, subsequent to breed formation.

In an attempt to perform a fair comparison between mixed breed and purebred dogs, we focused on nine largely recessive disease variants previously reported to exist in at least ten different pure breeds, and which were confirmed as common in both the mixed breed and purebred populations also in the present study. A prevailing view is that purebred dogs are more likely to be affected by inherited disorders (i.e., inherit a copy of the same recessive disease mutation from each parent) than mixed breed dogs as a consequence of the limited breed gene pool caused by genetic founder effects, intense selection, repeated inbreeding, and overuse of popular sires [32]. Conversely, random mating and outcrossing in mixed breed dogs may result in the health-increasing effects of hybrid vigor or heterosis through a reduced likelihood of both parents of a dog being carriers of the same recessive disorder. We found that mixed breed dogs were more likely to carry at least one of the evaluated disorders, but typically in a harmless heterozygous state, presumably due to the reduced likelihood of two carriers of the same condition having offspring. In contrast, purebreds were more likely to be genetically affected for one of the disorders included in the investigation. We thus provide the first comprehensive direct genotyping-based evidence suggesting that enrichment of recessive disease alleles in purebred dogs manifests as an overall higher proportion of the population being “genetically affected” due to homozygosity for a disease variant. Our findings supplement previous observations of breed dogs carrying significantly more predicted deleterious amino acid changing genotypes in the homozygous state relative to wolves [34].

Discovery of a disease-implicated variant on another genetic background does not equal confirmation that presence of the variant leads to disease onset. During recent years concerns have been raised by breeders, breed organizations and other community stakeholders regarding the use and marketing of DNA tests for breeds in which the finding has not been appropriately validated. Striving for increased harmonization within this field of DNA testing activities, we provide a suggested best practice protocol (Fig 3) for broader consideration by relevant stakeholders. In essence, detailed clinicopathological follow up studies are needed to ultimately confirm whether a variant is of relevance across breeds, and should be considered in veterinary care and breeding. We are currently pursuing a number of such separate investigations aiming to clinically validate the reported genetic findings (Table 3). Within the scope of this study, we provide case studies exemplifying how at least a number of disease variants are likely to lead to a similar disease phenotype on a mixed breed background. Awareness of inherited health risks also in mixed breed dogs is therefore warranted. For instance, providing dog owners and veterinarians with knowledge of a mixed breed dog’s MDR1 genotype status prior to anesthetic procedures ensures that appropriate medication protocols or adjusted medication doses can be used, thereby having a notable improvement in the dog’s clinical recovery and return to normal activities.

The wide variety of intriguing disease variant discoveries made in additional purebreds (in which the variant was not known to exist according to current literature) further confirms the utility of comprehensive panel screening as a research discovery, diagnostic, and breeding tool (Table 3). In particular, we significantly expand the number of breeds potentially affected by vWD type 1, *prcd*-PRA, factor VII deficiency, and HUU. We also provide a plausible genetic explanation for hereditary footpad hyperkeratosis (HFH) in additional terrier breeds; a potential cause of PRA in the Brussels Griffon (Griffon Bruxellois); and hypotheses to explore regarding cataract development in the Miniature Pinscher and potential bleeding propensity in the Bichon Frise. Intriguingly, we found a genetic variant previously described to cause a mild disproportionate dwarfism (skeletal dysplasia 2; SD2) in Labrador Retrievers [28] to be the predominant allele in Sussex Spaniels (14/17 analyzed individuals homozygous, and 2/17 heterozygous). The SD2 variant, and the mild phenotype associated with it (short legs with normal body length and width), represents a compelling putative explanation for short-leggedness in the Sussex Spaniel–a breed not reported to carry the common breed-defining chondrodysplasia variant in the *FGF4* gene [29]. Of final particular note, during preparation of this manuscript novel information emerged suggesting that the originally implicated risk variant for vWD type 2 [32] may in fact not be causal, and that another variant in the *VWF* gene may be the actual culprit [23]. This notion is supported by our findings of the originally published “risk variant” in more than 10 additional breeds, and by our clinical VWF measurements in dogs of several breeds at putative risk.

Modern genetic technologies now provide the means of generating “big data” to guide canine DNA testing priorities, breeding selections, breed health research, veterinary education and pet care. Turning big data into action is a challenge, which when hurdled is likely to have important implications for animal welfare. As one supportive tool for aiding this development, we announce the launch of the MyBreedData Canine Inherited Disorder Prevalence Database (http://www.mybreeddata.com). The aim of the database is to make breed-specific disease variant prevalence data publicly and easily accessible and amenable to regular updates. Such information advances breed health research, supports organizations establishing breeding guidelines (e.g., in determining whether a specific DNA test is relevant to the population in question and should be mandatory/optional for the breed), as well as advocates for openness on the part of DNA testing laboratories offering testing services to the breeder community regarding the true population relevance of a marketed test.

In conclusion, we report that risk variants for genetic disorders are prevalent in the general dog population, and confirm that mixed breed dogs may suffer from many of the same medical conditions as purebreds. Mixed breed dogs were more likely to carry a common recessive Mendelian disorder, while purebred dogs were more likely to be genetically at risk of one. Some disease variants still segregating in mixed breeds may have been eradicated or reduced in frequency through selective breeding in purebreds, while others are enriched in subsets of breeds or are breed-specific. We further expand knowledge on the distribution of known disease variants across purebreds through a number of genetic discoveries, paving the way for further clinical follow up investigations. Taken together, our findings emphasize the need for genetic testing as a tool to improve diagnostics, veterinary care, and ultimately the welfare of all dogs through informed breeding decisions. Going beyond disorders that can currently be tested for, all dogs—purebreds and mixed breed dogs alike—are likely to carry a number of unknown deleterious mutations. A holistic approach and destigmatization of healthy dogs carrying inherited disorders, combined with sustainable breeding practices maintaining population genetic diversity, is needed in the management of known disease mutations while avoiding the enrichment of novel unknown ones.

## Materials and methods

### Study sample

The mixed breed study sample (N = 83,220) consisted of customer samples originally submitted for breed testing on the Wisdom Panel platform (Wisdom Health, Vancouver, WA, USA) during February, 2015 –May, 2016. Any samples classified as F1 generation hybrid mixes of two breeds (e.g., “designer” dogs) by the breed testing platform were excluded in order to focus on truly mixed genetic backgrounds in the present study. The vast majority (92.6%) of the mixed breed study sample consisted of dogs from the United States (US), with the United Kingdom (UK; 3.3%) and Australia (2.3%) forming other notable subgroups (>1% of the sample). Noteworthy (>2% of estimated great-grandparents) representations of breed backgrounds in the mixed breed dogs were from American Staffordshire Terrier (10.0%), Labrador Retriever (7.0%), German Shepherd Dog (5.8%), Chow Chow (5.6%), Boxer (4.2%), Chihuahua (3.7%), Rottweiler (2.9%), Siberian Husky (2.8%), Australian Cattle Dog (2.6%), Golden Retriever (2.5%), Miniature Poodle (2.2%), and Beagle (2.1%). The breed composition of additional mixed breed dogs studied for clinical validation of signs of inherited disease was obtained using the commercially available Wisdom Panel breed ancestry test platform (Wisdom Health).

The purebred comparison sample (N = 18,102; S1 Table) consisted of dogs sampled at Wisdom Health (formerly Mars Veterinary) and Genoscoper Laboratories (Helsinki, Finland) between January, 2005 and October, 2016. Part of the sample (N = 6788) overlapped with dogs examined in our previous study [11]. The majority of tested dogs were from the US (44.1%), Finland (28.3%) and UK (11.4%), with other major subgroups formed by dogs from Germany (3.0%), the Netherlands (2.8%), Russia (2.2%), Australia (1.5%), and France (1.4%). For the purposes of this study, dogs were defined as “purebred” if registered according to established Fédération Cynologique Internationale (FCI), American Kennel Club (AKC), United Kennel Club (UKC), or the Kennel Club (UK) standards, with limited exceptions made for some true-breeding entities recognized by national registries. The purebred status of US dogs was additionally assessed using the Wisdom Panel platform (Wisdom Health). A total of 330 different breeds were included in the purebred study sample, with 224 breeds represented by ≥ 20 individuals. Breed inclusion in the study was independent of the number of disease variants known to exist in the breed *a priori*. The median contribution of an individual breed to the combined purebred study sample was 0.19% (min 0.006%; max 4.56%). In addition, archived samples from wild canids were genotyped (Gray Wolves, N = 65; Coyotes, N = 27; Dingos, N = 13).

Genetic analyses were carried out on DNA extracted from owner-collected, non-invasive cheek swab samples, or from blood/cheek swab samples collected at certified veterinary clinics in accordance with international standards for animal care and research. All dog owners provided consent for the use of their dog’s DNA sample for research purposes. Where applicable, drawing of blood samples was approved by the Animal Ethics Committee of the State Provincial Office of Southern Finland, Hämeenlinna, Finland (permit number: ESAVI/6054/04.10.03/2012).

### Genotyping

Genotyping of 152 disease variants (S2 Table) was carried out according to manufacturer-recommended standard protocols on a custom-designed Illumina Infinium HD genotyping bead chip targeting known point mutations, insertions, and deletions underlying canine inherited disorders ([11]; Illumina, San Diego, CA, USA). Marker selection was based on a review of known canine disease-implicated variants [1]. Measures taken to ensure high quality genotyping data included: 1) validation of individual disease assays with known control samples; 2) validation with synthetic oligonucleotides where no controls samples where available; 3) technical replicates of each disease assay; 4) manual review of genotype calls; and 5) a sample inclusion criteria requiring genotype calls for ≥98% of the analyzed markers. Genotypes from markers subject to patent- or license restrictions were stored only for research purposes, and not reported to dog owners.

Disease allele findings in additional purebreds were confirmed by standard capillary sequencing on an ABI3730xl DNA Analyzer platform (Thermo Fisher Scientific, Waltham, MA, USA) at the Sequencing Unit of the Finnish Institute of Molecular Medicine (FIMM). Preparation and purification of PCR-products for sequencing was carried out as previously described in detail [11] using ~20 ng of genomic template DNA and an Amplitaq Gold Master Mix-based protocol according to the manufacturer’s instructions (Applied Biosystems, Waltham, MA, USA).

### Statistical analyses

The statistical significance of differences between mixed breed dogs and the combined purebred study sample in the distribution of disease variants carried was evaluated with Pearson’s chi-square contingency table (2x2 and 2x3) tests evaluating observed vs. expected cell counts. For the analysis focusing on common recessive disorders carried in the heterozygous state, dogs carrying four disease variants were combined with dogs carrying three disease variants into a category of “≥3 variants carried” due to the limited number of individuals in the aforementioned category (N = 10). Similarly, in evaluation of common recessive disorders carried in the homozygous state, dogs carrying three disease variants (N = 1) were combined with dogs carrying two disease variants forming a category of “≥2 variants carried”.

### Clinical validation data

Medical background information and records on genetically affected dogs were collected through interviews with dog owners, veterinary clinicians, and breed club representatives. Public ophthalmological records were additionally accessed through the Finnish Kennel Club Registry Database [33]. Blood plasma samples were routinely collected at certified veterinary clinics, and submitted for vWF associated antigen (vWF:Ag) testing through either IDEXX Laboratories (IDEXX Europe B.V., Hoofddorp, The Netherlands) or the Veterinary Diagnostics Laboratory at the University of Utrecht, The Netherlands.

## Supporting information

S1 Table Study sample composition–purebred dogs and wild canids. (XLS)

S2 Table Genotyped genetic disease variants. (XLS)

S3 Table Shared and exclusive disease variants. (XLS)

S4 Table Prevalence-based ranking and frequencies of 152 disease variants in ~100,000 dogs. (XLS)
