# Correction: Frequency and distribution of 152 genetic disease variants in over 100,000 mixed breed and purebred dogs

**DOI:** 10.1371/journal.pgen.1007938

**Published:** 2019-01-18

**Authors:** Jonas Donner, Heidi Anderson, Stephen Davison, Angela M. Hughes, Julia Bouirmane, Johan Lindqvist, Katherine M. Lytle, Balasubramanian Ganesan, Claudia Ottka, Päivi Ruotanen, Maria Kaukonen, Oliver P. Forman, Neale Fretwell, Cynthia A. Cole, Hannes Lohi

The authors have provided an updated version of S2 Table. Please view the amended S2 Table below which includes the chromosome, position, reference allele, and alternate allele together with the reference genome assembly used for the analyses.

## Supporting information

S2 Table Genotyped genetic disease variants. (XLS)
